# Roles of dopamine receptor on chemosensory and mechanosensory primary cilia in renal epithelial cells

**DOI:** 10.3389/fphys.2014.00072

**Published:** 2014-02-26

**Authors:** Viralkumar S. Upadhyay, Brian S. Muntean, Sarmed H. Kathem, Jangyoun J. Hwang, Wissam A. AbouAlaiwi, Surya M. Nauli

**Affiliations:** ^1^Department of Pharmacology, The University of ToledoToledo, OH, USA; ^2^Department of Medicinal and Biological Chemistry, The University of ToledoToledo, OH, USA; ^3^Department of Pharmacology and Toxicology, College of Pharmacy, University of BaghdadBaghdad, Iraq

**Keywords:** mechanotransduction, fluid shear stress, mechanosensation, pharmacology, physiology

## Abstract

Dopamine plays a number of important physiological roles. However, activation of dopamine receptor type-5 (DR5) and its effect in renal epithelial cells have not been studied. Here, we show for the first time that DR5 is localized to primary cilia of LLCPK kidney cells. Renal epithelial cilia are mechanosensory organelles that sense and respond to tubular fluid-flow in the kidney. To determine the roles of DR5 and sensory cilia, we used dopamine to non-selectively and fenoldopam to selectively activate ciliary DR5. Compared to mock treatment, dopamine treated cells significantly increases the length of cilia. Fenoldopam further increases the length of cilia compared to dopamine treated cells. The increase in cilia length also increases the sensitivity of the cells in response to fluid-shear stress. The graded responses to dopamine- and fenoldopam-induced increase in cilia length further show that sensitivity to fluid-shear stress correlates to the length of cilia. Together, our studies suggest for the first time that dopamine or fenoldopam is an exciting agent that enhances structure and function of primary cilia. We further propose that dopaminergic agents can be used in “cilio-therapy” to treat diseases associated with abnormal cilia structure and/or function.

## Introduction

Dopamine is an endogenous catecholamine hormone that produces a range of effects in the nervous, immune, cardiovascular, and renal systems. Although classically produced in the brain and adrenal gland, dopamine is also biosynthesized in renal proximal tubules (Goldstein et al., 1972; Baines and Chan, 1980; Zimlichman et al., 1988). The five G-coupled protein dopamine receptors (DR) are categorized into D1-like (DR1 and DR5) and D2-like (DR2, DR3, and DR4) families (Sibley and Monsma, 1992). The role of dopamine in the renal system includes inhibition of Na^+^ reabsorption and blood pressure regulation through D1-like receptor activation (McDonald et al., 1964). Thus, the expression of both DR1 and DR5 in the kidney implies a profound role for both D1-like receptors in the renal dopaminergic system (Felder et al., 1984, 1989; Yamaguchi et al., 1993). However, the activation of DR5 in renal epithelial cells has not yet been studied.

Primary cilia are small membrane enclosed, hair-like structures that extend from the cell's apical surface. In renal tubules, cilia function as sensory organelles that initiate an increase in cytosolic calcium in response to tubular fluid-flow (Nauli et al., 2003). Cilia dysfunction perturbs calcium responsiveness and is causal to renal disorders such as polycystic kidney disease (PKD). Cardiovascular abnormalities including hypertension and left ventricular hypertrophy significantly contribute to mortality in PKD patients (Fick et al., 1995). Therefore, renal dopaminergic control of blood pressure remains an understudied aspect of ciliopathies such as PKD.

As chemosensory organelles, primary cilia depend on various receptors to be expressed on the ciliary membrane. This includes dopaminergic receptors, which are G-protein-couple receptors (Abdul-Majeed and Nauli, 2011). As mechanosensory organelles, primary cilia also depend on the expression of mechanosensitive molecules in the ciliary membrane. Some of these examples include mechanosensory polycystin-1 and sensory calcium channel polycystin-2 (Nauli et al., 2003). It is generally accepted that fluid-shear stress will bend primary cilia and activated polycystins complex. Activation of polycystins can induce calcium influx into cilioplasm (Jin et al., 2013). This ciliary calcium is further amplified through calcium-induced calcium release mechanism, resulting in an increase in the overall cytosolic calcium (Nauli et al., 2003; Jin et al., 2013).

As sensory organelles, primary cilia are viewed as compartments for sensory proteins (Nauli et al., 2011a,b). Thus, a larger compartment would assume to “house” more sensory proteins. As the result, it has been hypothesized that longer cilia tend to be more sensitive in chemo or mechanical-sensing. However, there was no hypothetical model that had shown this previously. Our studies thus showed the relationship between cilia length-function for the very first time in porcine kidney proximal tubule cells (LLCPK).

## Materials and methods

### Cell culture pharmacology

LLCPK cells plated on sterile glass-bottom plates were cultured to a confluent monolayer in Dulbecco's Modified Eagle Medium (DMEM) supplemented with 10% fetal bovine serum at 37°C in 5% CO_2_. Serum was then withdrawn from cells to allow differentiation. A faster differentiation could be achieved by cell-cell contact inhibiting cell growth and/or withdrawing serum from the cell culture media. Because only fully-differentiated cells have well-developed cilia, cell differentiation can be validated by immunostaining of the sensory proteins in the cilia or responsiveness of the cells to fluid-shear stress. We have previously discussed cell differentiation in details (Nauli et al., 2013). Once differentiated, dopamine or fenoldopam (SigmaAldrich, Inc.) were gently added to culture plates to achieve 1 nM to 10 μM final concentrations for 16 h in the absence of serum. For control experiments, vehicle alone was added to cells in the same manner and volume as those contained pharmacological agents.

### Immunofluorescence

Immunostaining was performed before and after flow experiments. Cells were fixed for 10 min (4% paraformaldehyde/2% sucrose in PBS) and permeabilized for 5 min (10% triton X-100). Primary antibodies were incubated for 1 h at the following concentrations: acetylated α-tubulin at 1:5000 (SigmaAldrich, Inc.), DR5 at 1:500 (EMD Millipore, Inc.). Cells were then incubated with fluorescent-conjugated secondary antibodies (1:500) as indicated in the text and mounted with DAPI hard set mounting media (VectorLabs, Inc.).

### Cilia length measurements

Primary cilia are composed of acetylated microtubule structures and were measured by direct immunofluorescence with acetylated α-tubulin staining (Abdul-Majeed and Nauli, 2011). Through calibrated image acquisition and analysis using MetaMorph software, primary cilia length was recorded. For statistical purposes, 40 cilia were measured for each treatment group.

### Cytosolic calcium measurements

Cells were incubated with Fluo2-AM (5 μM; 30 min; 39°C). Cells were then washed of residual dye and acclimated to allow complete ester hydrolysis. Cells were observed under a 40× objective lens with a Nikon Eclipse TE2000-U microscope controlled by Metafluor software. Cytosolic calcium was observed by recording calcium-bound Fluo2 excitation fluorescence at 488 nm and emission at 515 nm, as previously described (Nauli et al., 2008; AbouAlaiwi et al., 2009; Muntean et al., 2010; Abdul-Majeed and Nauli, 2011). Baseline calcium was observed for 10 min prior to data acquisition and then cells were subjected fluid-shear stress as indicated in the text. After each experiment, the maximum calcium signal was obtained by perfusion of ionomycin (10 μ M final concentration) followed by addition of EGTA (2 mM final concentration) to observe maximum and minimum calcium signals for normalization purpose. Conditions for all experiments were maintained at 37°C and 5% CO_2_ in an environmental chamber (In Vivo Scientific, Inc.).

### Perfusion

Cells were grown on customized culture plates to allow enable perfusate influx and efflux (AbouAlaiwi et al., 2009; Aboualaiwi et al., 2014). Fluid-shear stress was then applied to cells utilizing an Instech P720 peristaltic pump. We have previously calculated that kidney tubules experience shear-stress in a range of 0.2 to 20 dyne/cm^2^, depending on the rates of urine production (Nauli et al., 2006). To reflect physiological conditions, perfused fluid was therefore pumped into the cell culture dish and retained at shear stress of 0.8 dyne/cm^2^.

### Statistical analysis

Cilia length measurements consisted of *n* = 40 for each experimental group. Cytosolic calcium measurements consisted of *n* = 3 for each treatment. All data are reported as mean ± standard error of mean with statistical power greater than 0.8 at *p* < 0.05. Data were then analyzed utilizing ANOVA test followed by Tukey post-test. Analysis of data was performed with Prism GraphPad 5 software.

## Results

**Figure 1:**
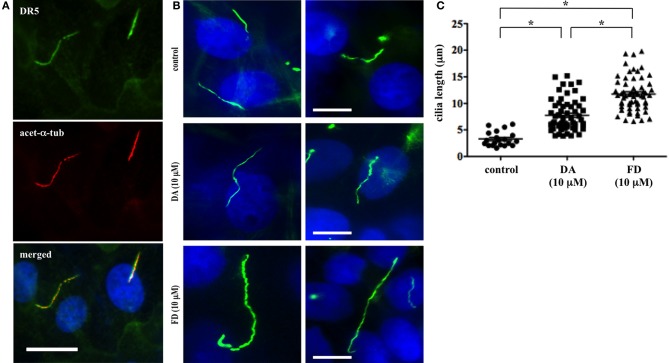
**Activation of DR5 increases primary cilia length of renal epithelial cells. (A)** Dopamine receptor type 5 (DR5; green) was localized to primary cilia of LLCPK cells. The cells were counterstained with ciliary marker acetylated α-tubulin (acet-α-tub; red), and nuclear marker (dapi; blue). Bar = 10 μm. **(B)** Dopamine and fenoldopam increased length primary cilia in renal epithelial cells. Primary cilia were observed with ciliary marker, acetylated α-tubulin, in LLCPK cells. After treated with mock (control), dopamine (DA), or fenoldopam (FD) for 16 h, cells were fixed and cilia length was observed and measured with fluorescence microscope. Bar = 5 μm. **(C)** Cilia length was significantly longer in epithelial cells treated with dopamine or fenoldopam. Cilia measurement was made from 4 preparations in each group, and at least 10 cilia were randomly selected in each preparation. The graph shows individual values of cilia length from measurements made in cells treated with mock (control), dopamine (DA), or fenoldopam (FD). Asterisks denote significant difference at *p* < 0.05. *N* = 40.

Primary cilia are critical sensory organelles, and cilia dysfunction inhibits renal cells from responding to fluid-flow (Nauli et al., 2003). Although the chemosensory roles of cilia have been proposed (Goetz and Anderson, 2010), most studies have focused only on developmental signaling pathways. Thus, much less is known about the structure-function relationship regarding primary cilia in the kidney. We previously showed that dopamine-receptor type 5 (DR5) localized to primary cilia in endothelial cells (Abdul-Majeed and Nauli, 2011). In this study, we discovered that DR5 also co-localized to the primary cilia in kidney proximal tubule cells (LLCPK) (Figure 1A).

To study the dopaminergic effect on primary cilia in LLCPK cells, we measured cilia length through immunofluorescent staining (Figure 1B). The average cilia length was observed to be 3.3 ± 0.3 μm. When treated with 10 μ M dopamine, cilia length was significantly increased to 7.8 ± 0.7 μm within 16 h. As dopamine is a general activator of D1-like dopamine receptors (DR1 and DR5), we next used fenoldopam to activate ciliary DR5. After a 16-h treatment with 10 μ M fenoldopam, cilia length was significantly increased to 11.8 ± 0.7 μm within 16 h. Data analysis of cilia length (*n* = 40) revealed statistically significant differences amongst control vs. dopamine, control vs. fenoldopam, and dopamine vs. fenoldopam (Figure 1C).

**Figure 2:**
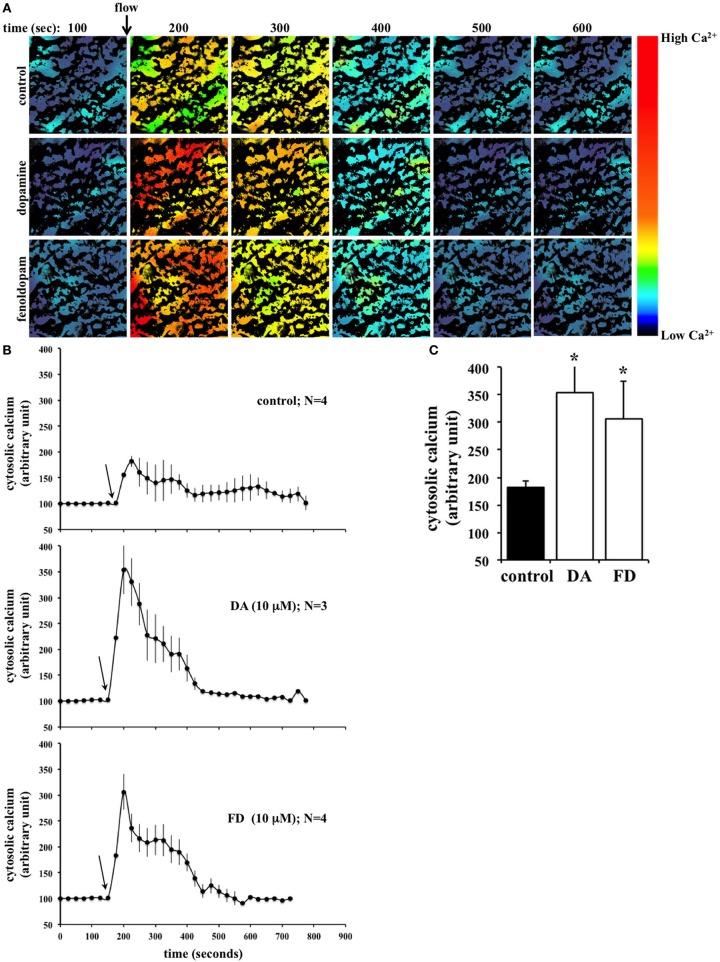
**Flow-induced calcium signaling is significantly greater in epithelial cells treated with dopamine or fenoldopam. (A)** Fluid-shear induced calcium signaling in LLCPK renal epithelial cells. Representative fluorescent images from each treatment are shown. Time is indicated in seconds (s). Arrow indicates the start of fluid-flow. Color bar indicates calcium level, where black-purple and yellow-red colors represent low and high calcium levels, respectively. **(B)** Cilia function was assessed from 3 to 4 preparations in each group, and at least 10 cells were randomly selected in each preparation. After treated with mock (control), dopamine (DA), or fenoldopam (FD) for 16 h, cells were challenged with fluid-shear stress. Arrows indicate the start of fluid-flow. **(C)** Peak calcium changes were averaged in cells treated with mock, dopamine, or fenoldopam. Asterisks denote significant difference at *p* < 0.05. *N* = 3.

Primary cilia in renal epithelium respond to fluid-flow by initiating an influx of extracellular calcium (Nauli and Zhou, 2004). We thus measured cytosolic calcium by incubating cells with calcium-specific indicator, Fluo2 and recording the calcium-bound emission signal. To mimic physiological conditions, cells were then subjected to fluid-shear stress at 0.8 dyne/cm^2^ while fluorescence emission was continuously recorded (Figure 2A). To examine the effect of increased cilia length on calcium influx, cells were treated for 16 h with dopamine or fenoldopam prior to loading with Fluo2. Pre-treatment with either dopamine or fenoldopam was found to significantly enhance the cellular calcium response to fluid-shear stress compared to control cells (Figure 2B). There was no statistical difference between dopamine and fenoldopam treated groups (Figure 2C).

**Figure 3:**
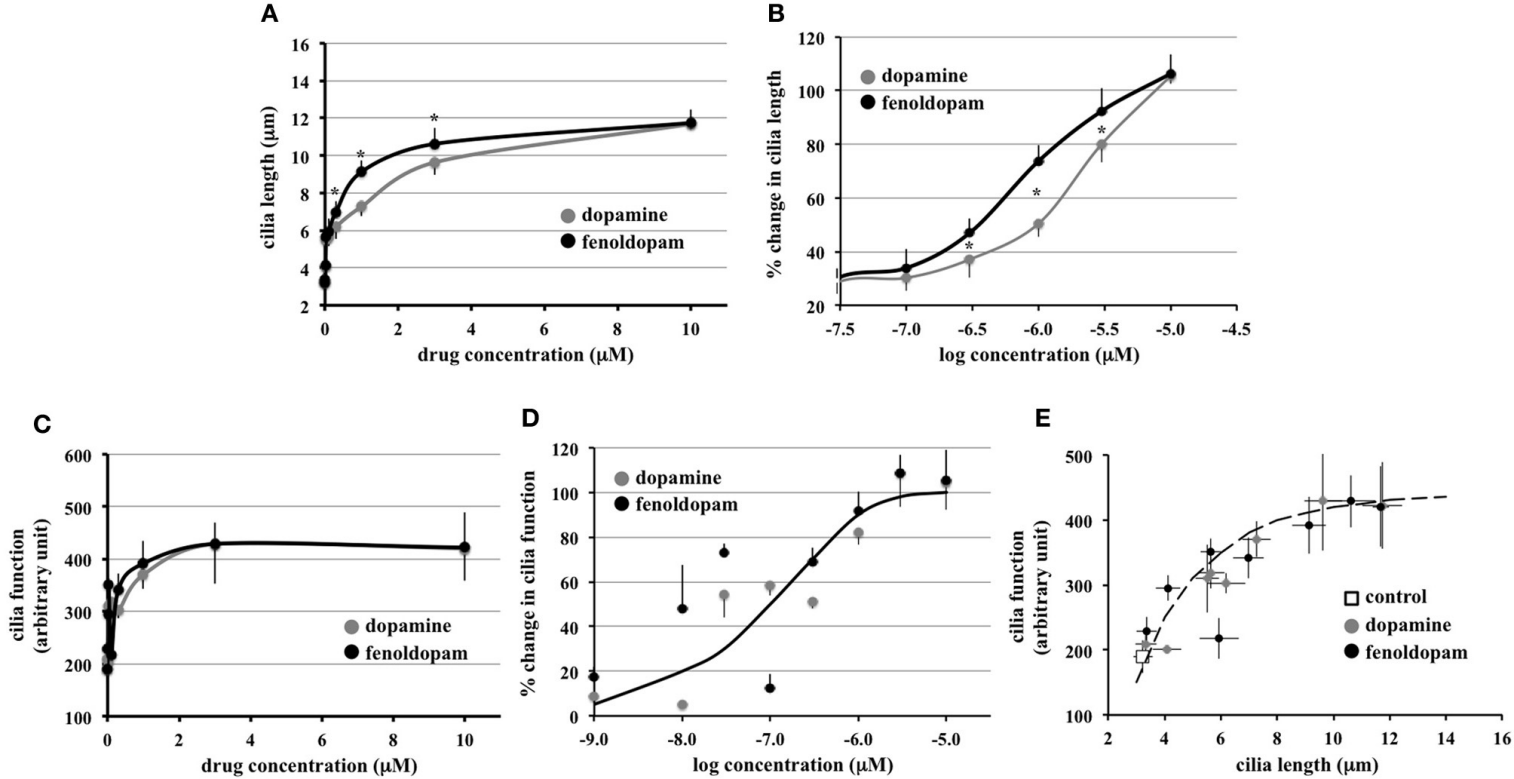
**Function of primary cilia as sensory organelles depends on the length of cilia. (A)** Hyperbolic curves indicated a positive correlation between cilia length and dopamine or fenoldopam. Averaged cilia length is shown from cells treated with dopamine or fenoldopam at 1 nM to 10 μ M. **(B)** Sigmoidal curves were generated with logarithmic transformation to indicate a cause-and-effect relationship between cilia length and dopamine or fenoldopam. **(C)** Hyperbolic curves indicated a positive correlation between cilia function and dopamine or fenoldopam. Cilia function was demonstrated by calcium signaling induced by fluid-shear stress. Averaged cilia function is shown from cells treated with dopamine or fenoldopam at 1 nM to 10 μ M. **(D)** Sigmoidal curves were generated with logarithmic transformation to indicate a cause-and-effect relationship between cilia function and dopamine or fenoldopam. **(E)** Peak calcium changes were averaged in cells treated with mock (control), dopamine, or fenoldopam at 1 nM to 10 μ M. A hyperbolic curve indicates a positive correlation between cilia length and function. Asterisks denote significant difference at *p* < 0.05. *N* = 28 for each dopamine or fenoldopam treatment.

To enable us to study cilia length-function relationship, we next examined the association between cilia length and cilia function, in terms of calcium influx in response to fluid-flow. We performed more robust logarithmic or graded dopaminergic-induced responses on cilia length (Figure 3A). In dose-response curve, effect of fenoldopam was more potent than that of dopamine (Figure 3B). Similarly, we performed logarithmic or graded responses on cilia function (Figure 3C). Based on the sigmoidal-relationship curve, we observed no difference in the efficacy or potency between dopamine and fenoldopam (Figure 3D).

Through extension of our graded-response curves, an increase in cilia length was observed to correlate with cilia function (Figure 3E). The graded response in dopaminergic-induced cells demonstrated a relationship between cilia length and cilia function, as indicated by the hyperbolic curve. Thus, this analysis revealed that cilia function is correlated to cilia length.

## Discussion

**Figure 4:**
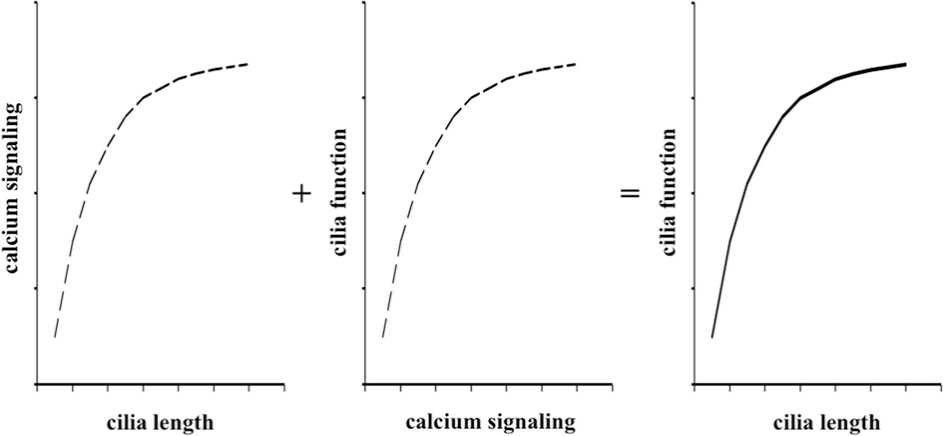
**Cilia length and function relationship is reflected by calcium signaling curves.** The roles of fenoldopam or dopamine on cellular responses to fluid-shear stress is a composite of interaction among intracellular calcium signaling, cilia length, and cilia function. The independent functions of pharmacological agents on length-calcium and calcium-function can thus be summed algebraically to yield cilia length-function relationship.

In the present studies, we used logarithmic concentrations of both dopamine and fenoldopam to facilitate us to understand the relationship between cilia length and function (Figure 4). These curves are classical pharmacological means to understand effects of pharmacological agents on cellular responses (Nauli et al., 2005). In the present study, we hypothesized that dopaminergic agents altered cilia structure and function. Because calcium signaling within a cilium has been shown to alter cilia length (Abdul-Majeed et al., 2012; Jin et al., 2013) and cytosolic calcium has been demonstrated to reflect cilia function (Nauli et al., 2008), we generated a model that guided us to understand cilia structure-function via calcium signaling in response to dopaminergic agonists. We thus propose that dopaminergic agonist can modulate cilia function by extending cilia length.

Endogenous dopamine neurotransmission signaling regulates many homeostatic functions (Sibley and Monsma, 1992). Since the discovery that the renal system biosynthesizes and utilizes dopamine independent of the nervous system, there has been significant interest in understanding the renal dopaminergic system (Carey, 2013). In regards to the renal system, dopamine has been shown to increase natriuresis through prevention of tubular Na^+^ reabsorption by activation of D1-like DR (Hughes et al., 1986). Thus, it has been proposed that the renal dopaminergic system plays an important role in systemic blood pressure regulation through DR1 and DR5.

PKD is one example of renal disorders that results in hypertension. In healthy renal tissue, primary cilia respond to fluid-flow by initiating an increase in cytosolic calcium. In PKD, on the other hand, primary cilia dysfunction leads to a reduction of intracellular calcium (Nauli et al., 2003). Further, PKD is the very first hypertensive disorder to be associated with abnormal primary cilia (Nauli et al., 2008; AbouAlaiwi et al., 2009). Although both D1-like dopamine receptors (DR1 and DR5) have been identified in proximal tubules, there has not been much work focusing on DR5 in renal dopaminergic signaling. Our study presents data to support the role for DR5 in renal dopaminergic signaling and cilia function. This may also help explain the clinical efficacy of fenoldopam, which is currently used as a treatment option for essential hypertension (Carey et al., 1984). Thus, by increasing cilia length fenoldopam is able to increase cilia function which may ultimately influence systemic blood pressure.

In this study, we show for the first time that DR5 localized to primary cilia in renal proximal epithelia. When D1-like receptors were activated by treatment with dopamine, the length of primary cilia was significantly increased. By selectively activating ciliary DR5 with fenoldopam, cilia length was further increased compared to both control and dopamine treatment. This data suggests that ciliary DR5 may contribute to more specific regulation on primary cilia than other DRs.

In response to fluid-flow, intracellular calcium levels were found to be increased after cells were treated with either dopamine or fenoldopam. As the perfusate did not contain pharmacological agents, the increased sensitivity to fluid-flow can be attributed to the increase in cilia length. Therefore, by increasing cilia length, overall cilia function was increased as well. To further test this hypothesis, intracellular calcium was graphed as a function of cilia length, which resulted in a positive correlation. This suggests that in our experiments, cilia length could modulate cilia function.

The ciliary role of DR5 is of further significance given the fact that D1-like receptors are expressed in low level in renal proximal tubules (O'Connell et al., 1998). Thus, the primary cilium may be an important central compartment that concentrates dopaminergic signaling, more specifically for DR5. Without doubt, there are many more interesting future experiments brought up by our studies. For examples, the numbers of receptors in the longer cilia will be needed to confirm. The mechanism by which dopaminergic agonist induced longer cilia needs to be further understood. An *in vivo* model may further shed some light into translational importance of our studies. Overall, our current studies provide evidence for the first time that dopamine or fenoldopam enhances both the structure and function of primary cilia.

### Conflict of interest statement

The authors declare that the research was conducted in the absence of any commercial or financial relationships that could be construed as a potential conflict of interest.
